# Failure characteristics of unsaturated intact loess under different hydraulic pathways

**DOI:** 10.1371/journal.pone.0334874

**Published:** 2025-10-31

**Authors:** Weiye Fu, Shengjun Shao, Aizhong Luo, Tao Li, Zijun Zhao

**Affiliations:** 1 School of Civil and Architectural Engineering, Xi’an University of Technology, Xi’an, Shaanxi, China; 2 School of Civil and Architectural Engineering, Guizhou University of Engineering Science, Bijie, Guizhou, China; 3 School of Mechanics and Civil Engineering, China University of Mining and Technology (Beijing), Haidian, Beijing, China; Islamic Azad University Mashhad Branch, IRAN, ISLAMIC REPUBLIC OF

## Abstract

This study systematically investigates the effects of two hydraulic pathways—wetting followed by loading (W-L) and loading followed by wetting (L-W)—on the water retention and strength characteristics of intact loess from a Xi’an metro line. Using an improved unsaturated triaxial testing system, experiments were conducted under controlled suction, net confining pressure, and shear stress levels. The Van Genuchten model accurately describes the water retention behavior, with the saturation-suction ratio (s/*S*_c_) exhibiting a linear relationship. The Critical State Line (CSL) for the L-W pathway exhibits a lower slope than that for the W-L pathway, indicating a reduction in shear strength and that hydraulic pathways strongly influence the suction contribution to loess strength. A threshold line in the *q-p*^’^ plane is identified, suggesting that hydraulic effects must be considered when the pre-wetting stress state exceeds this threshold. Scanning electron microscopy (SEM) analysis combined with quantitative pore analysis reveals that W-L induces pore expansion and cement dissolution, while L-W promotes particle compaction, partial cement fragmentation, and a measurable refinement of the pore network.

## 1. Introduction

Loess, a globally distributed Quaternary sediment, profoundly influences engineering practices in regions such as northwestern China [1,2]. Its unsaturated mechanical behavior is governed by matric suction, which affects deformation and strength [3,4]. While prior studies have focused on collapsibility [5,6], the role of hydraulic pathways—sequences of stress and suction changes—remains underexplored [7,8]. The hydraulic path dependence of loess has garnered increasing attention. Studies on remolded loess have demonstrated that hydraulic pathways induce distinct strength and deformation responses [9]. Furthermore, the microstructural evolution of intact loess during wetting-induced collapse has been recently revealed [10]. However, a systematic experimental comparison of how these specific hydraulic pathways govern the mechanical behavior and critical state of intact loess, which its natural structure and cementation is still lacking. This study systematically examines these pathways using advanced triaxial testing to bridge the gap between theoretical understanding and engineering applications. Most existing studies have focused on the influence of a single hydraulic path on the strength of loess. Beyond laboratory observations, the critical influence of hydraulic pathways is starkly evident in field-scale engineering failures. Case studies of tunnel collapses and slope failures in loess regions repeatedly highlight the triggering role of specific sequences of stress change and water infiltration. For instance [11], documented how slope instability was precipitated not merely by intense rainfall (wetting), but by the preceding excavation (loading) that created a critically stressed state. Similarly [12], analyzed tunnel lining failures attributed to the localized saturation of the surrounding loess after the completion of excavation and support installation. However, there is still a gap in the systematic comparison of the two paths of W-L and L-W. While previous studies on remolded loess have established the influence of hydraulic pathways on mechanical response, this study provides a systematic experimental comparison of the W-L and L-W pathways on intact loess using an improved triaxial apparatus. The results reveal significant differences in the resulting critical state lines between the two paths and identify a threshold line in the *q*-*p*’ plane that delineates the stress conditions where hydraulic path effects become critically important. Analysis of soil microstructure characteristics enables not only the establishment of a coupling relationship between macroscopic soil behavior and microscopic information, but also the revelation of the internal mechanisms governing soil property evolution. Scanning electron microscopy (SEM) has been widely used by researchers because of its imaging capability, high resolution, imaging performance, wide detection range and simple sample preparation process, and has become an important technical means to analyze the microstructure characteristics of different types of soil. By visualizing the arrangement of soil particles, pore structure characteristics and cement distribution, the technique provides a theoretical basis for interpreting the evolution of soil physical, chemical and biological characteristics [13–16].

This paper aims to explore the effects of different hydraulic pathways on the water retention and strength characteristics of intact loess through experimental research. By improving a fully automatic unsaturated triaxial apparatus, the study investigates the vertical strain and saturation of intact loess samples under controlled suction and net confining pressure conditions, comparing the effects of two hydraulic pathways: W-L and L-W. The research integrates macroscale mechanical testing with microstructural analysis using scanning electron microscopy (SEM) to unravel the underlying mechanisms of hydraulic path-dependent behavior. Specifically, SEM imaging at ×1000,  × 2000 magnification is employed to characterize particle arrangements, cementation dissolution patterns, and pore network evolution, which are critical for interpreting the observed strength differences. Mathematical models based on the Van Genuchten framework are established to quantify water retention behavior, while critical state analysis in the q-p’ plane identifies threshold conditions for hydraulic path effects. The findings not only provide a theoretical basis for engineering practices in loess regions but also contribute to the refinement of multiscale constitutive models that account for microstructure-mechanical property relationships.

## 2. Experimental apparatus and sample preparation

**Fig 1:**
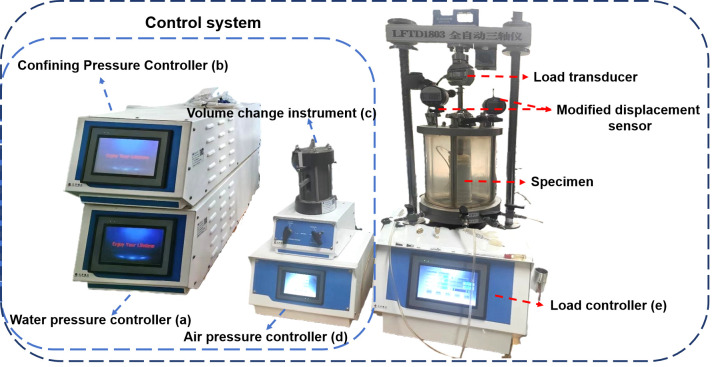
Improved fully automatic unsaturated triaxial apparatus (a) a water pressure controller (b) a confining pressure controller (c) a volume change meter (d) an air pressure controller (e) a load controller.

Fig 1 shows the improved fully automatic unsaturated triaxial apparatus, which consists of five main components: (a)a water pressure controller (b)a confining pressure controller (c)a volume change meter (d)an air pressure controller (e)a load controller. The apparatus controls suction balance (*u*_a_-*u*_w_) using the water and air pressure controllers and incorporates an independent stress path module to maintain specified stresses under controlled matric suction. A technical note is that the water pressure output is maintained slightly below the air pressure to prevent waterlogging of the internal proportional valve in the air pressure controller, which is crucial for ensuring its long-term measurement precision. The load controller measures the vertical displacement of the sample during testing (calibrated before each test). To ensure data accuracy and precise control, two displacement sensors (range: 50 mm, accuracy: 0.01 mm) were installed above the pressure chamber to measure sample displacement.

The load controller measures the vertical stress applied to the sample. Two displacement sensors were mounted on independent brackets above the triaxial cell to accurately measure the vertical strain of the sample. This setup was specifically implemented for the different hydraulic pathways. During the experiment, to maintain a constant deviator stress, the loading system activates the compensation mechanism that lifts the position of the triaxial cell. The inner displacement sensor of the apparatus is not optimized for capturing this specific movement. The displacement sensors provide a precise and direct measurement of the resultant vertical strain, ensuring data accuracy under constant stress conditions. Volume change was monitored through the volume change instrument, and radial strain was not directly measured but was derived from the vertical strain and volumetric strain data under the assumption of isotropic deformation, a common approach in unsaturated triaxial testing [17]. The improved apparatus features an enhanced digital control system that independent control of air and water pressures, enabling precise suction control throughout the testing process. The system’s pressure controllers have an accuracy of ±0.01 kPa, while the volume change measurement system has a resolution of 1 mm^3^.

The pressure controllers and volume change instrument were calibrated against standard references. To ensure measurement consistency, the vertical stress measurement system was calibrated using a certified proving ring, while the displacement measurement system was verified using precision gauge blocks. Further details aboutthis apparatus can be found in [18]. The testing program included duplicate tests for critical stress path conditions to verify result reproducibility. The maximum variation in measured strength parameters between duplicate tests was less than 5%, demonstrating repeatability of the experimental methodology.

The intact loess samples were obtained from a construction site for a Xi’an metro line at depths between 3–5 m. To preserve the natural structure and minimize disturbance, undisturbed block samples were carefully extracted using a manual excavation method after mechanical excavation reached the required depth. The blocks were meticulously trimmed by hand, immediately wrapped with multiple layers of plastic sealing film, and secured with light-proof tape to prevent moisture loss and mechanical damage. The intact loess block samples used in this study were obtained from a construction site of a Xi’an metro line. Site access and sampling were coordinated with the on-site project management team. No specific permits were required for this study as the sampling involved available construction materials from a standard geotechnical investigation and did not occur in a protected area or involve endangered species.

In the laboratory, the sealed blocks were first used for determination of basic physical properties including water content, density, and Atterberg limits. Cylindrical specimens (Φ39.1 mm × H80 mm) for triaxial testing were then carefully trimmed from the blocks using a manual lathe and trimming tools, with particular attention to preserving the natural fabric and minimizing mechanical disturbance during the preparation process. Table 1 presents the basic physical properties of the test material.

**Table 1:** Physical parameters of test material.

Natural gravimetric water content *w*_0_ (%)	23.2
Natural void ratio *e*_0_	1.05
Dry density *ρ*_*d*_ (g/cm^3^)	1.31
Plastic limit *w*_*p*_	21.6
Liquid limit *w*_*l*_	31.7
Plasticity index *I*_*p*_	10.1

## 3. Testing procedures

### 3.1. Different hydraulic path test for controlled suction

**Fig 2:**
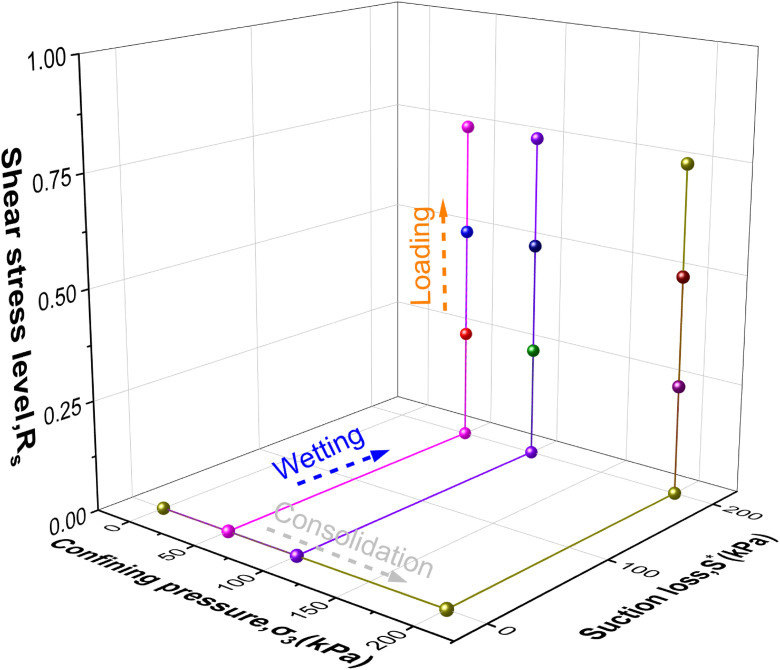
W-L path.

**Fig 3:**
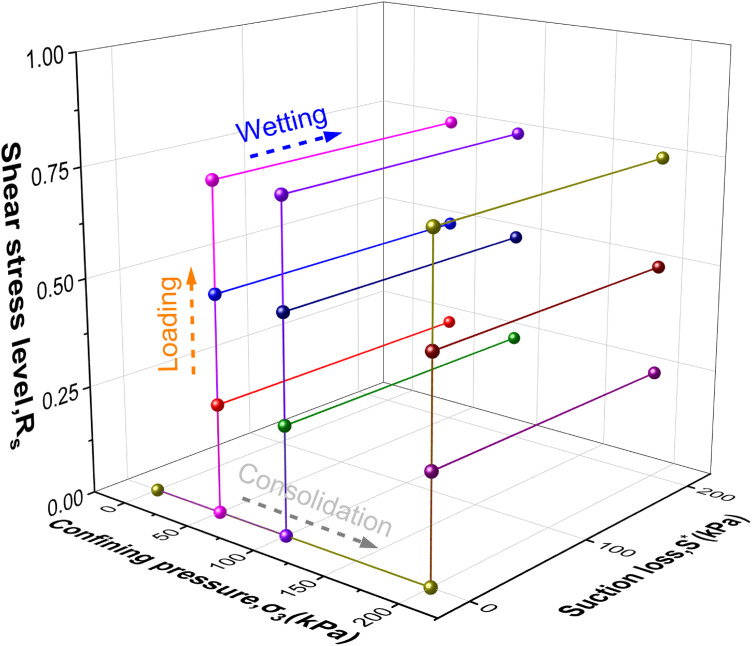
L-W path.

To explore the influence of different hydraulic pathways on intact loess, controlled suction and net confining pressure were applied during consolidated drained triaxial shear tests. Two hydraulic pathways were tested: W-L and L-W. The stress paths are illustrated in Figs 2 and 3, with net confining pressures of 50, 100, and 200 kPa and shear stress levels ($R_{s}=q/q_{f}$ , $q=\sigma_{1}-\sigma_{3}$) of 0.25, 0.50, and 0.75.

In the experiment, two pathways were implemented by placing the undisturbed specimen into the pressure chamber and securing it above the base. The water pressure controller (a) was used to regulate the inflow and outflow of water through the ceramic disk, while the air pressure controller (d) applied air pressure from the back pressure port. Both controllers were operated simultaneously to wet the specimen to saturation. The specific procedures for the two hydraulic pathways were as follows: For the W-L path: The specimen was first wetted under a constant net confining pressure and zero deviator stress until the target saturation was achieved. Only after this wetting stage was completed, the deviator stress was increased incrementally under drained conditions until the failure criterion (15% axial strain) was reached. For the L-W path: The specimen was first loaded under a constant matric suction and net confining pressure to a predefined shear stress level (*R*_s_). Then, while maintaining the deviator stress and net confining pressure constant, the matric suction was reduced incrementally. The specimen was sheared to failure during this wetting process, with failure defined as either reaching 15% axial strain or exhibiting catastrophic collapse under a specific suction value. The suction at failure (*s*_f_) was recorded for each test.

### 3.2. SEM test

In order to deeply explore the deformation mechanism of intact loess under different hydraulic pathways, scanning electron microscopy (SEM) imaging technology was used in this study to analyze the microstructure of intact loess after mechanical tests. In order to ensure the representativeness and accuracy of the microscopic observation results, the characteristic parts of all samples were selected as the observation areas after different hydraulic path tests. At the same time, in order to obtain detailed microstructure information (such as particle arrangement, cement state and pore evolution characteristics), × 1000 and ×2000 magnifications were used for systematic observation.

### 3.3. Quantitative microstructural analysis

**Fig 4:**
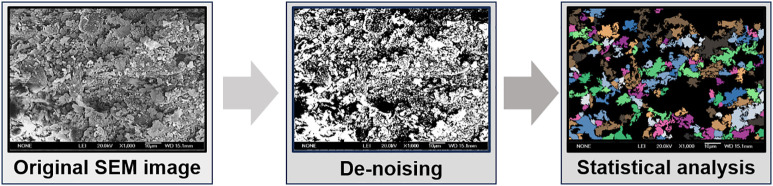
PCAS analysis flowchart.

To obtain semi-quantitative metrics from the SEM images, a pore size distribution analysis was performed using the Pore and Crack Analysis System (PCAS) software. The processing procedure involved: (1) importing the original SEM image; (2) applying image de-noising and filtering techniques to enhance feature clarity; (3) converting the image to a binary format using a consistent threshold to distinguish pores from particles; and (4) statistically analyzing the identified pores to classify them by equivalent diameter into macro-pores, meso-pores, small pores, and micro-pores based on established criteria [19]. This workflow is illustrated in Fig 4.

## 4. Experimental results and discussion

### 4.1. Water retention characteristics under different hydraulic pathways

**Fig 5:**
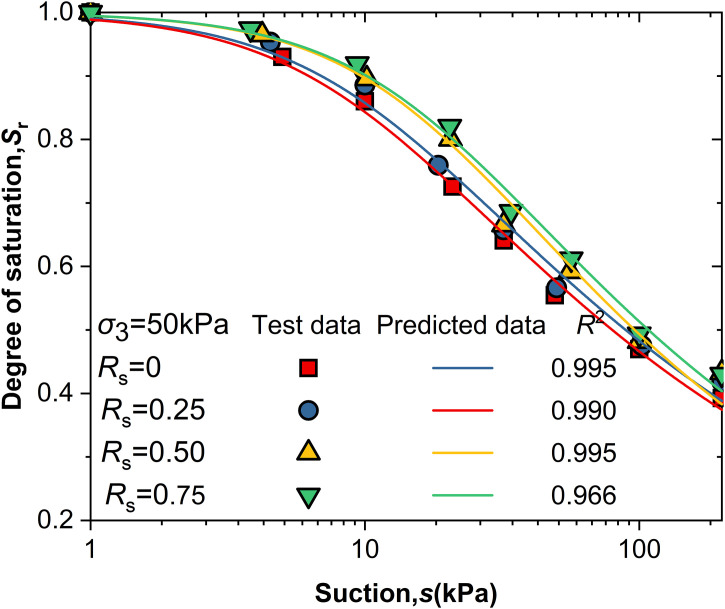
*Sr*-*s* curve under net confining pressure of 50kPa.

**Fig 6:**
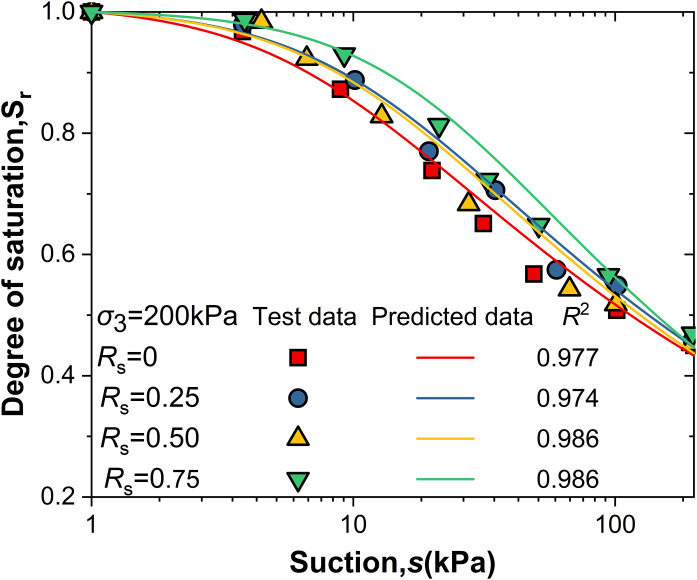
*Sr*-*s* curve under net confining pressure of 200kPa.

Figs 5 and 6 shows the relationship between suction (*s*) and saturation (S_r_) under different shear stress levels (*R*_s_) and net confining pressures. The curves exhibit a steep initial rise followed by a gradual increase, with higher R_s_ values shifting the curves upward. The Van Genuchten model [20] was used to describe the water retention characteristics of intact loess, with the Sr expression given by Equation (1). The model parameters (α, n, m) were determined using the least squares method, and the air-entry value (*S*_c_) was identified as the Intersection of the asymptote of the log *S*_r_ - log *s* curve and *S*_r_ = 1.

Its formulation has been widely validated for loess, and it provided an excellent fit to the study experimental data (*R*^2^ > 0.96), supporting its use under the hydraulic paths applied in this study. This study employs the Van Genuchten(V-G) model to describe the water retention characteristics of intact loess. The expression for *S*_r_ is given by:


$$S_{r}=[1+(\alpha s{)}^{n}{]}^{-m}$$ (1)

The parameters n and m in the equation are determined by the least squares method, under the assumption that the asymptote of the logSr-logs curve intersects with *S*_r_ = 1 at the air entry value *S*_c_ (*S*_c_ = 1/ α) [21]. Based on the V-G model fitting parameters under different conditions of and Rs in Table 2, and the comparison between the measured data and the model-predicted data in Fig 5 σ₃ = 50kPa and Fig 6 σ₃ = 200kPa, the V-G model effectively predicts the water retention characteristics during wetting within the suction range studied.

**Table 2:** Wetting sample parameter table.

$\sigma_{3}$ /kPa	*R*_s_	*q/kPa*	*S*_*f*_	*S*c*/kPa*	α /kPa^-1^	*n*	*R* ^2^
50	0	0	0	4	0.101	1.326	0.995
50	0.25	59	0	10	0.091	1.325	0.991
50	0.50	118	21	16	0.059	1.385	0.962
50	0.75	177	76	20	0.057	1.367	0.967
100	0	0	0	6	0.099	1.293	0.990
100	0.25	91	0	13	0.087	1.301	0.984
100	0.50	182	16	18	0.057	1.332	0.969
100	0.75	273	65	23	0.051	1.347	0.968
200	0	0	0	10	0.095	1.272	0.986
200	0.25	129	0	15	0.079	1.297	0.974
200	0.50	258	6	21	0.056	1.301	0.986
200	0.75	387	95	27	0.046	1.361	0.977

**Fig 7:**
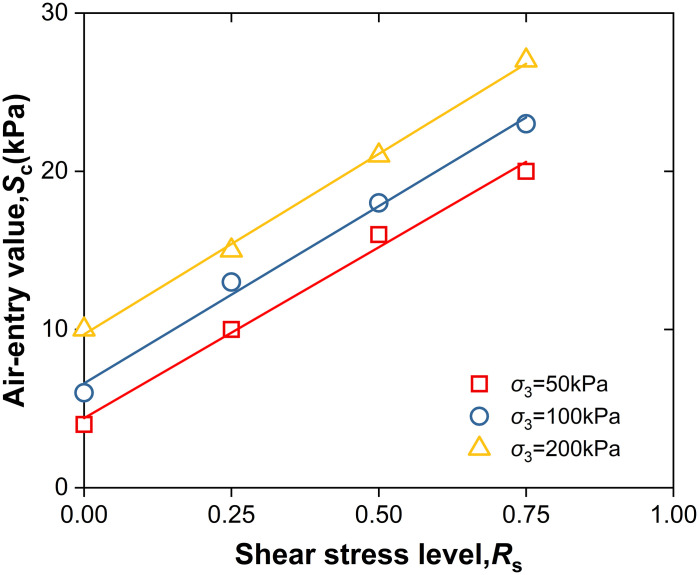
*S*_c_-*R*_s_ Relationship line.


As summarized in Table 2, and it is shown in Fig 7 that the intake value *S*_c_ is linearly correlated with the shear stress level *R*_s_. With corresponding numerical values provided in Table 3. This relationship can be empirically expressed as:

**Table 3:** *S*_c_-*R*_s_ parameter table.

$\sigma_{3}$ /kPa	*S*_c0_	*C*_1_	*R* ^2^
50	4	21.6	0.992
100	6	22.4	0.992
200	10	22.8	0.998

$$S_{c}=S_{c0}+C_{1}R_{s}$$ (2)

Here, *S*_c0_ represents the air-entry value corresponding to a shear stress level of *R*_s_ = 0kPa, and *C*_1_ denotes the soil parameter characterizing the influence of shear stress level on the air-entry value. The fitted data are summarized in Table 3.

Substituting Equation (2) into Equation (1) leads to


$$S_{r}=\{1+[s/(S_{c0}+C_{1}R_{s}){]}^{n}{\}}^{-m}$$ (3)

**Fig 8:**
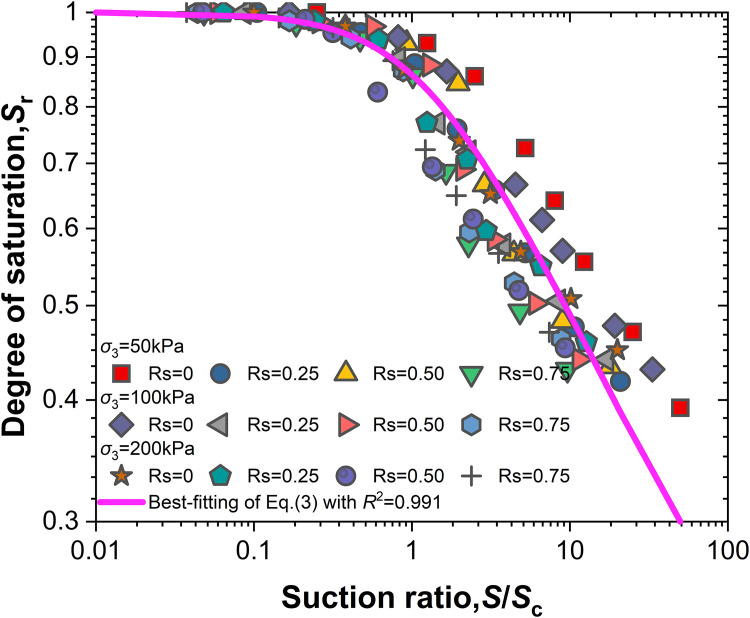
Sr~s/Sc relationship curve.

Equation (3) describes the relationship curve between the change in saturation and the suction ratio (*s*/*S*_c_) (the ratio of suction (*s*) to air-entry value (*S*_c_), as shown in Fig 8. The experimental data points are concentrated within a narrow band and exhibit a very strong linear correlation (*R*^2^ = 0.991), as shown by the best-fit line in Fig 8. This high coefficient of determination indicates that the relationship between saturation (*S*_r_) and the suction ratio (*s*/*S*_c_) can be approximated by a straight line, independent of the shear stress level (*R*_S_). This further confirms that the shear stress level has a negligible influence on the normalized water retention behavior during wetting.

### 4.2. Strength characteristics under different hydraulic pathways

#### 4.2.1. Stress-strain characteristics of different hydraulic pathways.

**Fig 9:**
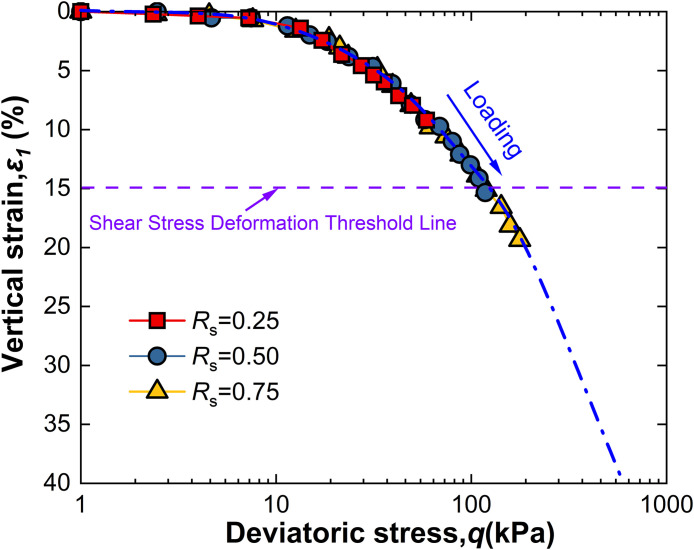
*ε*_1_-q curve of W-L at 50kPa.

**Fig 10:**
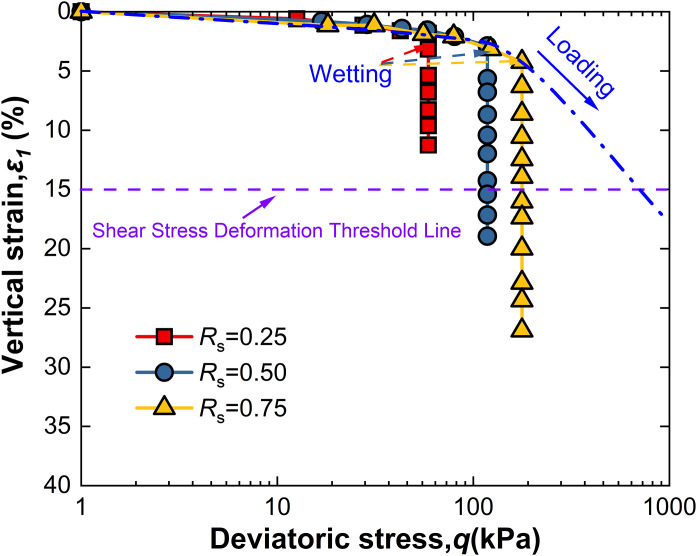
*ε*_1_-q curve of L-W at 50kPa.

**Fig 11:**
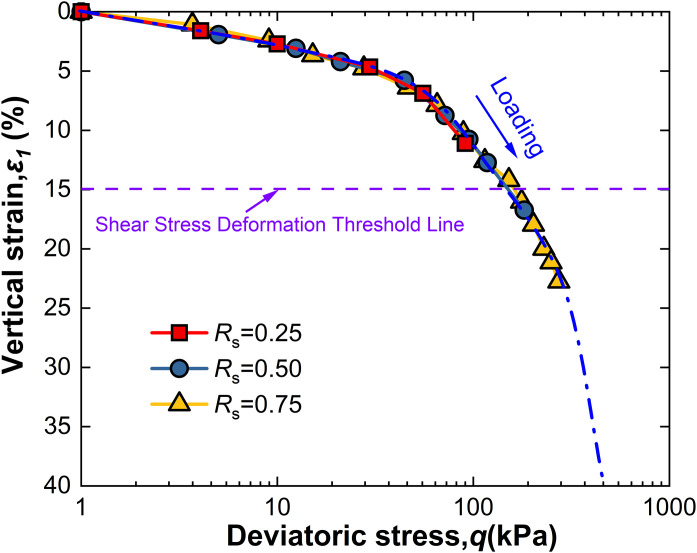
*ε*_1_-q curve of W-L at 100kPa.

**Fig 12:**
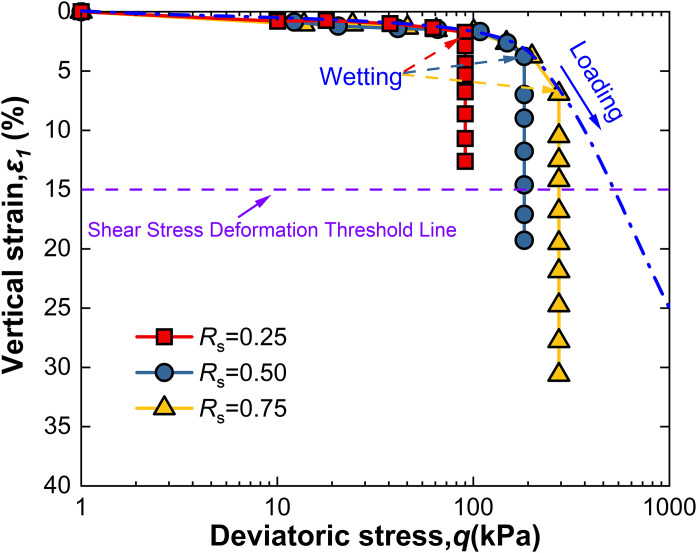
*ε*_1_-q curve of L-W at 100kPa.

**Fig 13:**
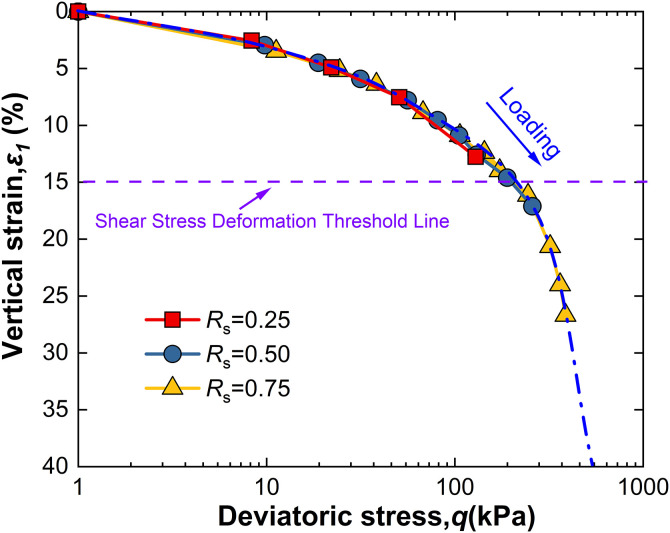
*ε*_1_-q curve of W-L at 200kPa.

**Fig 14:**
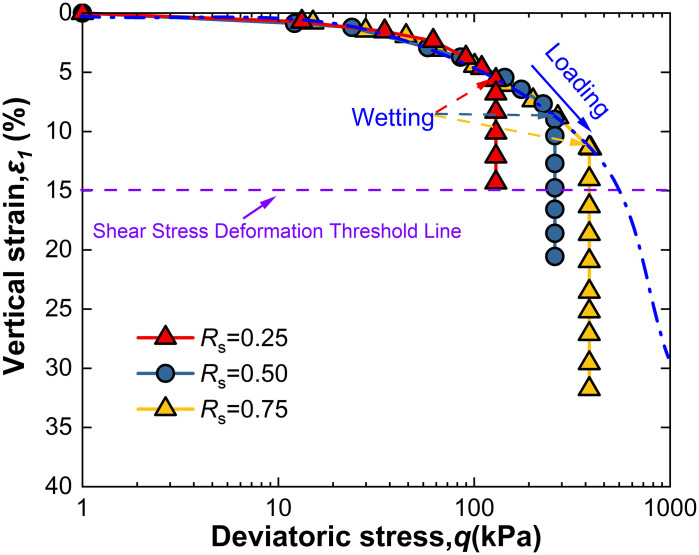
*ε*_1_-q curve of L-W at 200kPa.

For intact loess, two hydro-mechanical paths are compared through experiments: one where samples are first wetted to saturation and then loaded to a specified shear stress level *R*_s_, and another where samples are first loaded to *R*_s_ and then wetted to saturation. The strength characteristics at failure under these two conditions are observed as shown in Figs 9–14.

Observation of Fig 9 reveals that for intact loess samples first saturated by wetting and then subjected to a specified shear stress level *R*_s_, vertical strain increases with increasing deviatoric stress, an effect that becomes more pronounced with increased net confining pressure. Moreover, at all levels of net confining pressure tested, *R*_s_ = 0.25 does not reach the failure criterion. This phenomenon suggests that the failure behavior of intact loess under the W-L path is significantly influenced by the shear stress level *R*_s_ and net confining pressure.

Observation of Fig 10 shows that when intact loess samples are first loaded to a specified shear stress level *R*_s_ and then wetted to saturation while controlling suction *s*, vertical strain initially increases slowly with deviatoric stress. As suction *s* decreases, vertical strain increases monotonically, an effect also becoming more pronounced with increased net confining pressure. Furthermore, at all levels of net confining pressure tested, *R*s = 0.25 does not meet the failure criterion; however, other conditions reached failure before suction dropped to 1 kPa, with their *s*_f_ values recorded in Table 2. This indicates that the failure behavior of intact loess under the L-W path is significantly affected by the shear stress level *R*_s_, net confining pressure, and suction *s*. These observations highlight both similarities and differences between the two different hydro-mechanical paths.

#### 4.2.2. Strength characteristics under different hydraulic pathways.

**Fig 15:**
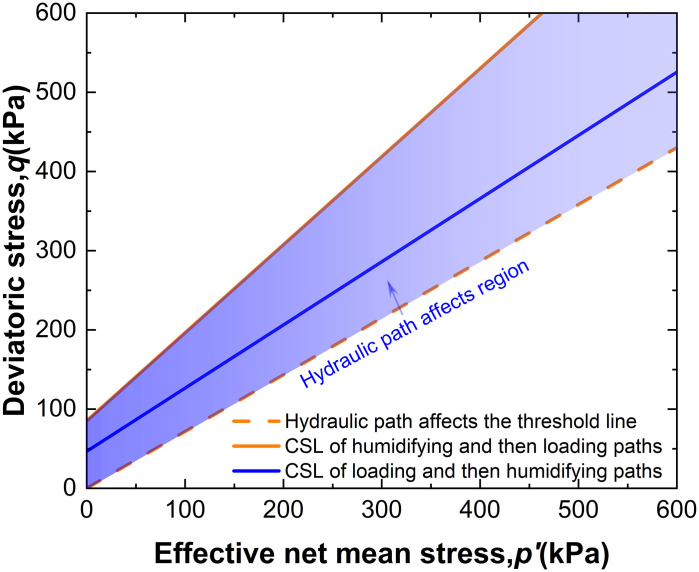
CSL line in the q-p’ plane.


In this study, the intact loess was tested with a failure criterion set at *ε*_1_ = 15%. The failure stresses (*p*_f_, *q*_f_) under net confining pressures of 50kPa, 100kPa, and 200 kPa were determined (where net mean stress *p* = *q*/3+ $\sigma_{3}$). The failure stresses obtained by substituting the experimental results from Figs 9 and 10 into Equation (4) were plotted on the *q-p’* plane, as shown in Fig 15. Here, p′ [22] is expressed as follows:

$$p^{\prime }=p+S_{r}s$$ (4)

Here, *p*′ represents the effective net mean stress, *S*_r_ is the degree of saturation, and *s* is suction.

In the *q-p*′ plane, under constant deviator stress *q*, the loss of suction leads to a reduction in the effective net mean stress. For the path of L-W, failure occurs before the suction is completely lost(*s*_f_ ≠ 0), indicating that the hydraulic path has a direct influence on the intact loess.

It is observed that the experimental data points for both hydraulic pathways are distributed within a narrow region and can be approximated by straight lines. The slope of the fitted line for the W-L path is *K*_H_ = 1.112, while for the L-W path, it is *K*_L _= 0.798. This suggests that the failure characteristics of intact loess under both hydraulic pathways can be described using the Mohr-Coulomb strength theory.

The Critical State Line (CSL) for the L-W path lies below that determined for the W-L path. Since failure occurs in the L-W path before reaching full saturation, it is evident that the contribution of suction to the strength of intact loess is influenced by the hydraulic path. Previous studies that considered only wetting without accounting for the hydraulic path may lead to misjudgment of the shear strength of loess, thereby affecting engineering safety.

At low *R*_s_ levels, intact loess does not fail under either path. However, at high *R*_s_ levels, failure occurs in the L-W path when wetting reaches *s*_f_, whereas in the W-L path, failure occurs at full saturation. This indicates the existence of a hydraulic path influence zone and a critical shear stress level threshold *R*_cr_. When the shear stress level *R*_s_ exceeds the threshold *R*_cr_, the influence of the hydraulic path on the strength of intact loess must be considered, which has significant implications for practical engineering applications.

**Fig 16:**
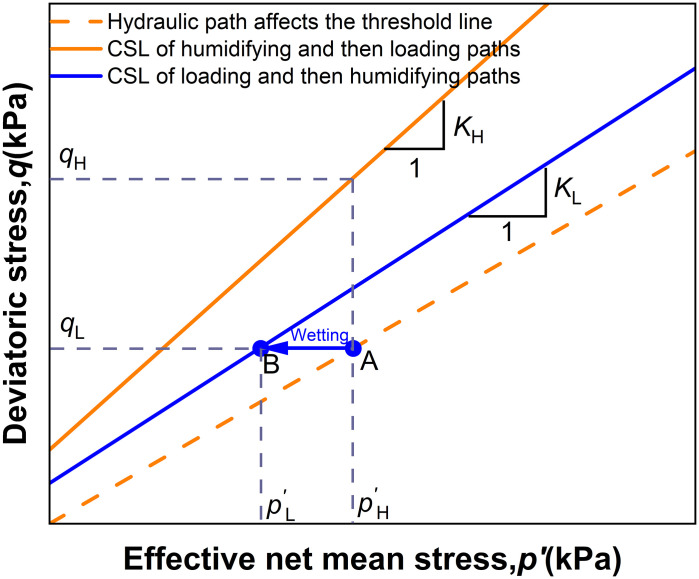
q-p’ in-plane analysis line.


In Fig 16, point A (*p*_H_′, *q*_H_) represents the natural suction state. When moistening from point A to point B (*p*_L_′, *q*_L_), assuming the suction at point B is *s*_f_, the path AB is described, wherein, the saturations before moistening are denoted as $S_{r0}$, $s_{0}$.

$${p_{H}}^{\prime }=\frac{q_{H}}{K_{H}},{p_{L}}^{\prime }=\frac{q_{L}}{K_{L}}=\frac{q_{H}R_{cr}}{K_{L}}$$ (5)

Among them, $R_{cr}=\frac{R_{SL}}{R_{SH}}=\frac{q_{L}}{q_{H}}$, by introducing the influence factor *ξ* (defined as the ratio of the critical state line slopes, *ξ* = K_L_/K_H_, representing the strength reduction factor induced by the hydraulic pathway) and utilizing Equation (5), we obtain:


$$R_{cr}=\xi (1-\frac{S_{r0}s_{0}-S_{rf}s_{f}}{p^{\prime }})$$ (6)

Where ξ =0.718 and *s*_0_ = 200kPa, the threshold line for the hydraulic path in the q-p plane is determined according to Equation (6). For enhancement to the saturated state, *S*_rf_ = 1 and *s*_f_ = 0. When the pre-enhancement stress state is above the threshold line, the effect of hydraulic action on the intact loess must be considered. The threshold line in the q-p’ plane (Fig 16) is proposed as a linear criterion. This simplification is empirically derived from our experimental data, which show a linearly increasing threshold stress with confining pressure, and is justified for its practicality in geotechnical engineering design.

The complete critical state line equations, derived from linear regression, are:


$$W-L\;pathway:\;q\;=\;1.112\;p^{\prime }\;+\;46.62\;kPa\;(R^{\text{2}}\;=\;0.98)$$ (7)

$$L-\;W\;pathway:\;q\;=\;0.798\;p^{\prime }\;+\;85.25\;kPa\;(R^{\text{2}}\;=\;0.97)$$ (8)

To statistically quantify the difference between these lines, an Analysis of Covariance (ANCOVA) was performed. The analysis confirmed that the reduction in the CSL slope for the L-W pathway is highly statistically significant (*p* < 0.01), while the difference in the intercepts is not significant (*p* = 0.329). It demonstrates that the hydraulic pathway does not merely apply a constant strength offset (which would be reflected in the intercept). Instead, it fundamentally alters the frictional nature of the soil. The lower slope M directly corresponds to a lower critical state friction angle, indicating that the L-W pathway degrades the soil’s intrinsic frictional resistance. This mechanistic distinction, revealed by the statistical analysis, explains why the strength loss under L-W pathway becomes increasingly severe.

### 4.3. Microstructure characteristics

**Fig 17:**
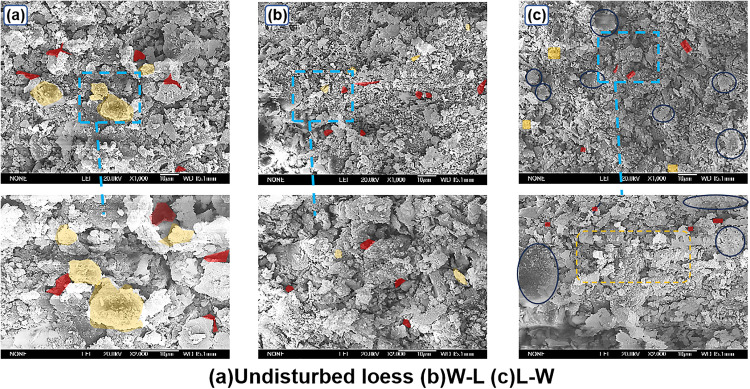
SEM images of hydraulic path under different multiples.

Through the analysis of the SEM images at ×1000 and ×2000 magnifications in the Fig 17, it can be known that: the integrity of the cementing material (yellow area) between the particles of the original loess is high, the particle arrangement has not been disturbed by the outside, and the pores (marked in red) are evenly distributed, presenting the typical cementation structure and pore characteristics of loess in its natural state; in the W-L path samples, the particles are significantly dispersed, the cementing material dissolves due to wetting, the pores expand and the connectivity increases, which conforms to the evolution mechanism of cementation failure and soil structure loosening caused by wetting; in the L-W path samples, the particles are closely arranged after loading, the pores are compressed and reduced, the cementing material is locally broken (yellow dashed box area) but not completely dissolved, and the deep blue circle area shows that some particles are compacted closely under stress, reflecting the characteristics of structural compression promoted by loading and subsequent local damage of the cementing material caused by wetting [23–25].

**Fig 18:**
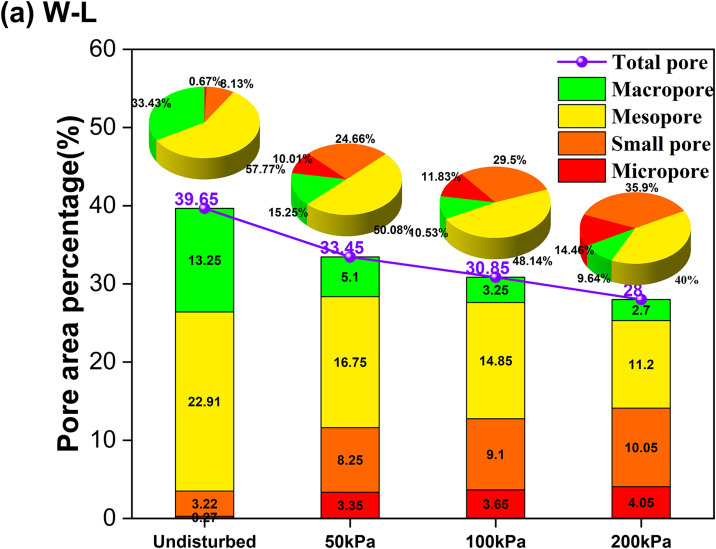
Results of the quantitative pore size distribution under the W-L pathway.

**Fig 19:**
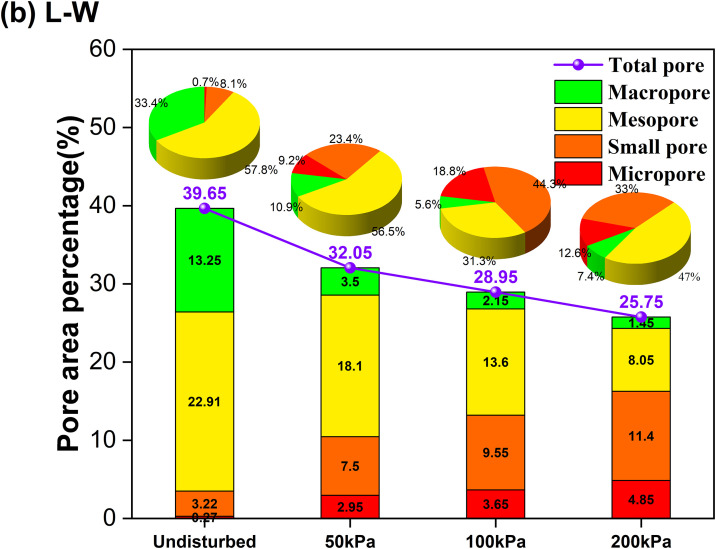
Results of the quantitative pore size distribution under the L-W pathway.

For the W-L pathway (Fig 18), the data indicates a pronounced decrease in the volume of macro-pores compared to the undisturbed loess. However, this is accompanied by a significant increase in the volume of meso-pores and small pores. This quantitative evidence suggests a mechanism whereby the intrusion of water weakens and destabilizes the large, metastable pores characteristic of intact loess, causing them to collapse and reorganize into a greater number of smaller, more homogeneously distributed pores. This breakdown of the natural soil fabric and the loss of its inherent structural strength directly contribute to the macroscopic strength reduction observed. For the L-W pathway (Fig 19), the data demonstrates an even more dramatic decrease in macro-pores and meso-pores, coupled with a sharp increase in the proportion of micro-pores. This provides conclusive quantitative evidence for the mechanism of extensive particle rearrangement and compaction under the applied load, which crushes and eliminates larger pores. The resulting microstructure is highly densified but is composed of a weakened assembly of finer pores and damaged cementation, which is directly linked to the severe reduction in the critical state friction angle observed in the macroscopic tests.

**Fig 20:**
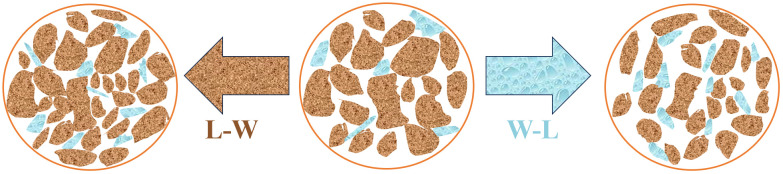
Schematic diagram of microscopic mechanism under different hydraulic paths.

Fig 11 shows the microstructure evolution mechanism of loess under different hydraulic pathways. The brown area represents the loess particles, and the blue area represents the liquid: the original loess shows the initial distribution of particles and water. Through the L-W path,the loading effect causes the particles to rearrange, arrange tightly, and improve the structural compactness. In the subsequent wetting, water exerts a local influence on the compacted structure. Through the W-L path,water intrusion weakens the intergranular cementation in the wetting stage,resulting in pore expansion, enhanced connectivity, and increased particle dispersion. Subsequent loading further aggravates the structural looseness. Fig 20 reveals the differential regulation mechanism of the Hydraulic path action sequence in the hydraulic path on the grain arrangement and pore evolution of loess from a microscopic scale, and provides a microscopic visual basis for explaining the differences in the macroscopic mechanical behavior of loess.

This study investigated two fundamental hydraulic pathways; however, field conditions often involve more complex hydraulic pathways, such as cyclic wetting-drying under load or infiltration to intermediate saturation levels. The marked differences in behavior between W-L and L-W paths suggest that cyclic or partial wetting-drying may induce cumulative fabric changes and accelerated strength reduction [25]. Further research into these complex pathways is necessary to develop constitutive models capable of predicting long-term performance of loess infrastructures under varying climatic conditions. This study provides the foundational understanding required for such advanced investigations.

## 5. Conclusions

This study investigated the water retention and strength characteristics of intact loess under different hydraulic pathways using an improved fully automatic unsaturated triaxial apparatus.

The Van Genuchten model effectively predicted the water retention characteristics of intact loess, and the relationship between saturation and suction ratio (*s*/*S*_c_) was approximated by a linear expression. The critical state line (CSL) under the L-W pathway was lower than that under the W-L pathway, highlighting the influence of hydraulic pathways on loess strength. A threshold line in the *q-p*’ plane was established, indicating that the hydraulic effect on intact loess should be considered when the stress state before wetting exceeds this threshold. These findings provide valuable insights for engineering practices in loess regions.

The W-L path results in the dissolution of cement, enhanced pore connectivity and obvious particle dispersion. Due to loading, the L-W path makes the particles more closely arranged, the pore structure is refined, and the cement is only partially broken. Based on these findings, engineering practice in loess regions should incorporate distinct strength parameters according to avoid detrimental hydraulic pathways, and employ the identified threshold line as an evaluation criterion for stability assessments under hydraulic path effects. Furthermore, these findings provide critical insights for constitutive modeling of unsaturated loess. The experimental results demonstrate that hydraulic pathway controls the mechanical response by defining distinct Critical State Lines. Therefore, constitutive models should incorporate hydraulic path dependence through path-dependent critical state parameters, a defined threshold stress criterion for hardening and softening rules, and microstructure evolution laws. These advancements will enable more accurate predictions of unsaturated loess behavior under complex hydro-mechanical loading.
